# Improved generation of anti-tumor immunity by antigen dose limitation

**DOI:** 10.1186/1476-8518-5-2

**Published:** 2007-02-09

**Authors:** Joshua D Shofner, Juan G Vasquez, Carole L Berger, Richard L Edelson

**Affiliations:** 1 Department of Dermatology, Yale University, 333 Cedar Street, New Haven, CT USA; 2 Yale Comprehensive Cancer Center, Yale University, 333 Cedar Street, New Haven, CT USA

## Abstract

**Background:**

The malignant cells of cutaneous T cell lymphoma (CTCL) display immunogenic peptides derived from the clonal T cell receptor (TCR) providing an attractive model for refinement of anti-tumor immunization methodology. To produce a clinically meaningful anti-tumor response, induction of cytotoxic anti-CTCL cells must be maximized while suppressive T regulatory cells (Treg) should be minimized. We have demonstrated that engulfment of apoptotic CTCL cells by dendritic cells (DC) can lead to either CD8 anti-CTCL responses or immunosuppressive Treg induction. Treg generation is favored when the number of apoptotic cells available for ingestion is high.

**Methods:**

In this study, we sought to determine whether the balance between immunity and immunosuppression could be shifted towards a CD8 anti-CTCL response by lowering the ratio of apoptotic CTCL cells available for DC ingestion. CTCL cell apoptosis was produced by engagement of the TCR by anti-CD3 antibody affixed to magnetic beads.

**Results:**

The physical perturbation inherent in passage through a separation column induced monocytes to differentiate into DC, demonstrated by increased expression of class II and CD86 and decreased expression of the monocyte marker CD14. The immature DC internalized and processed apoptotic CTCL cells and could potentially present the tumor-derived peptides in the context of MHC class I and II. As the number of apoptotic cells increased, there was a dose-dependent increase in the expression of Treg markers CTLA-4, CD25, and FoxP3, with a ratio of apoptotic cell/DC loading of > 10:1 corresponding to the greatest Treg induction. These inducible phenotypic Treg also functionally inhibited CD8-mediated perforin expression in vitro. At lower levels of apoptotic cell/DC loading of < 5:1, there was an expansion of the CD8 T cell compartment with increased perforin expression and increased CTCL cell death, indicating anti-tumor activity.

**Conclusion:**

These findings demonstrate that the ratio of apoptotic cells supplied to DC is an important determinant of whether CD8 anti-tumor immunity or immunosuppression is generated.

## Background

Cutaneous T cell lymphoma (CTCL) is an umbrella designation that unifies a diverse group of clinical presentations on the basis of histopathologic and immunologic criteria. The malignancy is a clonal proliferation of epidermotropic T cells [1-3], that uniformly carry a common T cell receptor (TCR) and also display cell surface expression of a memory (CD45RO^+^), inducer (CD4^+^), and cutaneous homing leukocyte antigen (CLA^+^) phenotype. Initially the tumor cells localize in the skin of afflicted patients, surrounding Langerhans cells that contribute to the CTCL cell growth [4]. As the disease progresses, the malignant cells become more poorly differentiated and often spread hematogenously throughout the body, as a leukemia forecasting a much poorer prognosis. It is the early epidermal focus of malignant T cells surrounding a central Langerhans cell [5], an immature member of the dendritic cell (DC) series [6], that is the diagnostic hallmark of the disease, the Pautrier microabscess [2]. CTCL tumor cells lack the co-stimulatory molecules required to trigger an immune response contributing to their ability to evade induction of anti-tumor immunity and thus, persist and disseminate. Despite the role of DC in providing proliferative support for the malignancy, DC immunotherapy has demonstrated clinical benefit in this disease [7,8].

We have recently found that passage of cells from CTCL patients through an anti-CD3 magnetic bead column can achieve the simultaneous induction of apoptosis in the malignant T cell population and differentiation in the monocyte population creating, after overnight culture, a population of tumor-loaded DC capable of initiating an immune response in vitro [9]. This column-based method of generating tumor-loaded DC utilizes the mechanistic principles discovered in extracorporeal photopheresis (ECP), the first FDA-approved immunotherapy for cancer [10]. Clinical trials of ECP have shown response rates of 73% in late stage CTCL patients with an extremely safe side effect profile [11]. ECP has been recently modified by the addition of an overnight incubation step. The new procedure has been termed " Transimmunization", wherein malignant T cells were rendered apoptotic by ultraviolet A (UVA) light photo-activated 8-methoxypsoralen (8-MOP) and were avidly engulfed by immature DC, whose transition from monocytes was triggered by the physical perturbation produced when the cells were passed through the UVA exposure plate of the ECP apparatus. In Transimmunization, the transitioning DC and the apoptosing leukocytes are co-cultivated overnight to permit engulfment of the apoptotic cells and subsequent processing and presentation of the tumor-derived peptides prior to re-infusion into the host [12].

We have previously shown that when CTCL cells encounter autologous DC loaded with high numbers of apoptotic cells, they adopt the phenotype and function of T regulatory (Treg) cells, expressing high levels of the Treg markers CTLA-4, CD25, and FoxP3, as well as secreting interleukin-10 (IL-10) and transforming growth factor-β (TBF-β) and suppressing normal T cell antigen driven secretion of interleukin-2 (IL-2) and interferon-γ(IFN-γ) [4]. The induction of Treg from responding CTCL cells may be hindering the effectiveness of existing immunotherapies for CTCL, and understanding the mechanism of their induction is paramount to the generation of more effective immunotherapy.

In these experiments, we sought to determine if the level of apoptotic cell loading controlled the balance between the development of an anti-tumor immune response and immunosuppressive Treg cell generation. We found that when lower numbers of apoptotic cells were processed by DC, a CD8 T cell response could be stimulated while at higher levels of apoptotic cell up-take by DC we could induce CTCL cell Treg conversion and inhibit the CD8 T cell response.

## Methods

### Patient Population

Specimens were obtained from a leukapheresis harvest of CTCL patients (in accordance with the guidelines of the Yale human investigation committee) undergoing treatment with standard ECP. All patients had advanced disease with clonal CD4^+ ^T cell populations present in the peripheral circulation as determined by immunophenotyping with antibodies to the clonotypic variable region of family-specific TCR or polymerase chain reaction to detect rearrangements of the beta or gamma chain of the TCR as well as CD8 T cell compartments > 10% of the circulating lymphocyte population.

### Cell Isolation

On Day 0, peripheral blood mononuclear cells (MNL) were isolated from the leukapheresis harvest by centrifugation over a ficoll-hypaque gradient followed by two washes in RPMI 1640 (Gibco, Gaithersburg, MD) containing 10% AB serum and 2 mM EDTA. An aliquot of the MNL was then isolated, purified, and cultured for subsequent re-addition on Day 1. Within this aliquot, CD4^+ ^and CD8^+ ^cells were column-purified using 40 μl Macs α-human CD4 and CD8 microBeads (Miltenyi Bioteck, Auburn CA) according to manufacturer's instructions. The enriched CD4^+ ^and CD8^+ ^T cells (> 97% ± 0.5% CD4^+ ^and > 98% ± 0.5% CD8^+^, respectively) were suspended in RPMI 1640 with 15% autologous serum and IL-2/IL-7 and cultured individually in separate wells of a 12 well tissue culture plate (Falcon). Another aliquot of the Day 0 isolated MNL was reserved to construct the apoptotic cell and DC dose response curves, and controls. MNL (2 × 10^7^) from the leukapheresis harvest were incubated with 40 μl Macs α-human CD3 microBeads following the manufacturer's directions. As previously described [9], CTCL cell binding to CD3 antibody rendered the malignant T cells apoptotic. As a control, another portion of MNL (2 × 10^7^) was incubated with 40 μl Macs α-human CD4 microBeads. Following the twenty minute incubation, the cells were passed through the magnetic bead column, allowing separation of the MNL into either CD3+ and CD3- fraction or CD4+ and CD4- fractions. The CD3-treated and CD4-treated cells were then cultured in 3 ml RPMI 1640 containing 15% AB serum. In some experiments, increasing doses of CD3-treated cells were added to 10^5 ^autologous DC obtained from the column eluate to create the dose response curves. Controls consisted of increasing doses of CD4-treated CTCL cells added to 10^5 ^autologous DC.

On Day 1, the individually cultured column-purified CD4 and CD8 cells (10^6^) were added to the CD3-treated apoptotic cell-loaded DC as well as the CD4-treated control population. After a second overnight culture, the cells were harvested, counted, and immunophenotyped for markers of T cells, DC, Tregs, and apoptotic cells.

### Immunophenotyping

In order to monitor enrichment of the purified CD4^+ ^and CD8^+ ^cell populations and to measure the effect of loading DC with differing number of apoptotic malignant T cells, the cells were stained by two-color immunofluorescence with a panel of antibodies to monocytes, DC, Tregs, and cytotoxic T cells. Cells (1 × 10^6^) were incubated with 10–20 μl of fluorochrome conjugated monoclonal antibody for 30 minutes in the dark at 4°C. The antibodies were directly conjugated to fluorescein (FITC) or phycoerythrin (PE) and included: CD3-FITC (pan T cell); CD4-FITC (inducer T cell); CD8-FITC (cytotoxic T cell); CD25-FITC (IL2 receptor); CD14-FITC (monocytes); HLA-DR-FITC (anti-class II MHC molecule) and CD86-PE (B7.2 co-stimulatory molecule) and their isotype controls. Cells were washed once and suspended in PBS and read on a FC500 flow cytometer (Beckman Coulter) within 24 hours.

Combined membrane and cytoplasmic staining was performed following manufacturers instructions for cell fixation and permeabilization (Intraprep kit, Beckman Coulter). Antibody combinations included: membrane CD4-FITC/cytoplasmic CTLA4-PE (inhibitory member of the co-stimulatory family); membrane CD3-FITC/cytoplasmic CTLA4-PE; membrane CD4-FITC/cytoplasmic FoxP3 (inhibitory transcription factor); membrane CD3-FITC/cytoplasmic Apo2.7-PE (apoptotic cells); membrane CD8-FITC/cytoplasmic perforin-PE (cytotoxic granule); and isotype controls (Beckman Coulter). Data was analyzed using the CXP software (Beckman Coulter).

### Statistical Evaluation

The expression of DC markers and the induction of a Treg or cytotoxic T cell response were evaluated statistically in 3 to 5 replicate cultures by the student's t test or if the data was not normally distributed the Mann-Whitney Rank Sum Test using the Sigma Stat analysis program.

## Results

### CTCL Cells Rendered Apoptotic by CD3 Antibody Binding

**Figure 1:**
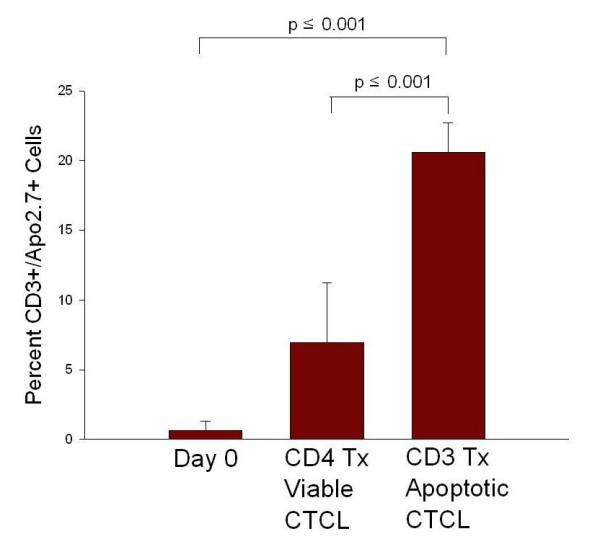
**Induction of CTCL cell apoptosis by anti-CD3 antibody**. CTCL cells were incubated for twenty minutes with either anti-CD4 or anti-CD3 antibody and cultured overnight with column-passaged monocytes. Apoptosis was determined on Day 1 by measuring percentage of cells co-expressing the T cell marker, CD3, and the early apoptotic marker, APO2.7. Tx = treated. Statistical analysis performed with student's t Test.

MNL including monocytes and CTCL cells obtained from a leukapheresis harvest of CTCL patients were passed through an anti-CD3 magnetic bead column to induce apoptosis in the antigen-experienced CTCL cells and were then co-cultured with column-passaged monocytes. As a control, an aliquot of the leukocytes was passed through an anti-CD4 magnetic bead column, and these viable CTCL cells were cultured with autologous DC in the same manner as the apoptotic T cells. In Figure 1, apoptosis was determined by measurement of the co-expression of the membrane T cell marker CD3 and cytoplasmic expression of the early apoptotic marker APO2.7. On primary Day 0 isolation, the percentage of CD3^+ ^cells expressing the apoptotic marker APO2.7 was 0.646%. After overnight culture, 6.96% of the T cell population bound to anti-CD4 antibody-conjugated beads was apoptotic. When lymphocytes were treated with CD3 antibody-conjugated beads, a 2.96-fold increase (P ≤ 0.001) in the percentage of apoptotic T cells was found (20.576%). Thus, CTCL cells treated with anti-CD3 antibody were rendered apoptotic at a significantly higher percentage and are termed apoptotic CTCL cells for all future experiments. Those CTCL cells treated with anti-CD4 antibody had a significantly higher percentage of viable cells and are denoted as viable for the remainder of the experiments.

### Passage of Monocytes through Magnetic Column Induces DC differentiation

**Figure 2:**
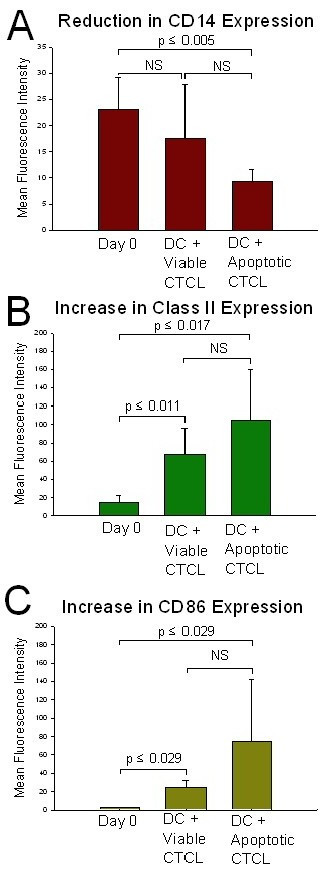
**DC differentiation from monocytes induced by column passage and apoptotic CTCL cell loading**. DC and apoptotic CTCL cells were co-cultured following passage of MNL through an anti-CD3 magnetic bead column. DC differentiation was monitored on Day 1 by measuring expression of cell surface markers using a flow cytometer gated on the monocyte population. **A. **Significant decrease in expression of monocyte marker CD14 was found on column-passaged monocytes co-cultured with apoptotic CTCL cells when compared to the level identified on Day 0 monocytes. **B. **Significant increase in expression of class II was found on column-passaged monocytes co-cultured with apoptotic CTCL cells when compared to the level identified on Day 0 monocytes. **C. **Significant increase in the expression of CD86 was found on column-passaged monocytes co-cultured with apoptotic CTCL cells when compared to the level identified on Day 0 monocytes Statistical analysis performed with student's t test or Mann Whitney Rank-Sum Test. Apoptotic = CD3-treated, Viable = CD4-treated, NS = not significant

Apoptotic CTCL cells were co-cultured with column-passaged monocytes and markers known to be up regulated during DC differentiation [12] were analyzed after overnight culture to determine the ability of passage through the magnetic bead column to drive the evolution of the monocyte population into DC. Monocyte differentiation into immature DC was measured by monitoring changes in expression of the monocyte marker, CD14, and DC markers, class II and CD86. Loss of CD14 expression was revealed by a decrease in the mean fluorescent intensity (MFI) of the CD14 fluorochrome from primary isolation on Day 0 followed by co-culture with viable CTCL cells. The maximum reduction in CD14 MFI was found when column-passaged monocytes were co-cultured with CTCL cells rendered apoptotic by CD3 antibody (Fig 2A). A 43% reduction of expression of the monocyte marker CD14 was found in comparison to the level expressed on monocytes isolated on Day 0 (P ≤ 0.005). There was no significant difference in the reduction in CD14 expression between differentiating DC cultured with viable CTCL cells or apoptotic CTCL cells. However, the addition of apoptotic tumor cells enhanced the decrease in CD14 expression while the presence of viable CTCL cells was not as effective in stabilizing the monocyte to DC conversion.

In addition, expression of class II and CD86 increased significantly (P ≤ 0.017–P ≤ 0.029 respectively) upon co-culture with column-differentiated DC fed apoptotic CTCL cells as compared to Day 0 controls. Class II expression increased from the level found on Day 0 monocytes to an intermediate increase found on the transitioning immature DC that had been co-cultured with viable CTCL cells (P ≤ 0.011) to the maximal increase seen when apoptotic CTCL cells (Fig. 2B) were co-cultured with the immature DC. A 5.5-fold increase in class II expression was found when the DC were fed apoptotic CTCL cells and compared to the monocytes isolated on Day 0. Expression of the co-stimulatory molecule CD86 also increased from day 0 to the intermediate increase found on transitioning immature DC that had been co-cultured with viable CTCL cells (P ≤ 0.029) to the maximal increase in DC fed apoptotic CTCL cells (Fig. 2C). A 25.8-fold increase in CD86 expression was found when DC that had engulfed apoptotic cells were compared to monocytes tested on primary isolation. Therefore, passage of CTCL cells through an anti-CD3 magnetic bead column and subsequent co-culture of newly apoptotic CTCL cells with column activated monocytes generated significantly enhanced DC differentiation. Co-culture of column-activated monocytes with viable CTCL cells was also effective in generating immature DC but the addition of apoptotic CTCL cells enhanced the adoption and stabilization of this phenotype.

### Induction of a Treg phenotype

**Figure 3:**
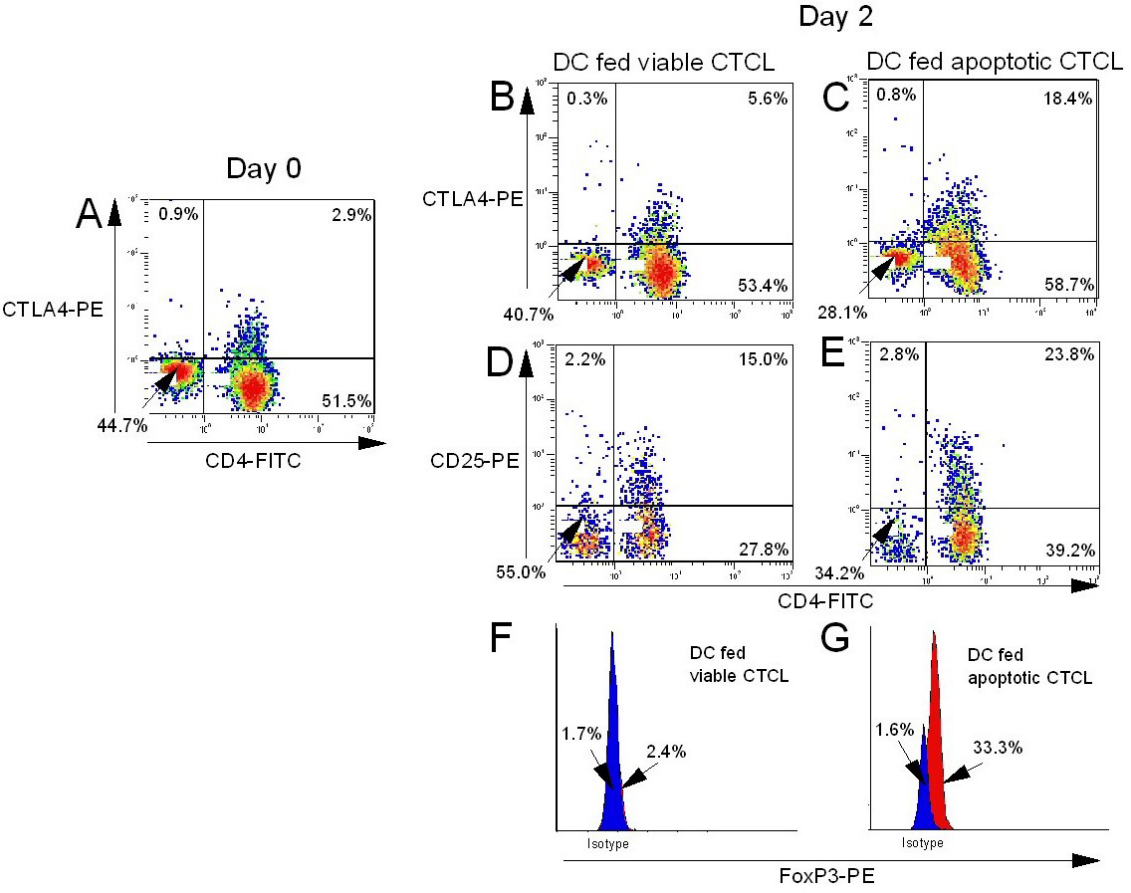
**Induction of a T regulatory phenotype in CTCL cells**. Untreated Day 0 CTCL cells were stained for co-expression of CD4 on the cell surface and CTLA-4 in the cytoplasm. DC were fed viable CTCL in ratio of > 10:1 or apoptotic CTCL cells in ratios that exceeded 10 viable (B, D, & F) or apoptotic CTCL cells (C, E, & G):1 DC and cultured overnight. Fresh responding CTCL cells were added on Day 1 and subsequently co-cultured. On Day 2, co-expression of: **B & C **membrane CD4 and cytoplasmic CTLA4; **D & E **membrane CD4 and membrane CD25; or **F & G **cytoplasmic expression of FoxP3 were measured. Results are representative of 5 separate experiments performed on cells isolated from cultures of 5 different CTCL patients. Apoptotic = CD3-treated, Viable = CD4-treated

Freshly purified CTCL cells were incubated overnight with DC that had ingested large numbers of apoptotic CTCL cells using an apoptotic CTCL cell to DC ratio of ≥ 10:1. A portion of the responding CTCL cell population adopted the phenotype of T regulatory cells, expressing the cell surface marker CD25 and the cytoplasmic markers CTLA-4 and FoxP3. CTLA-4 was up regulated in the cytoplasm of CTCL cells exposed to autologous DC that had engulfed large numbers of apoptotic cells (Fig. 3C), as compared to the control group (Fig. 3B) and the primary day 0 isolate (Fig 3A). CTCL cell membrane expression of CD25 increased upon co-culture with autologous DC that had engulfed large numbers of apoptotic CTCL cells (Fig 3E) as compared to controls (Fig. 3D). The viable CTCL cells were also partially activated to express increased membrane CD25 (Fig 3D), but not other markers of Treg conversion (Fig 3B &3F). Cytoplasmic expression of FoxP3 increased 13.9-fold following incubation with autologous DC that had engulfed large numbers of apoptotic cells (Fig. 3G) as compared to the control population (Fig. 3F). Thus, by co-culturing freshly purified CTCL cells with autologous DC that have engulfed large numbers of apoptotic CTCL cells, the responding CTCL cells adopt a phenotype specific for Treg cells.

**Figure 4:**
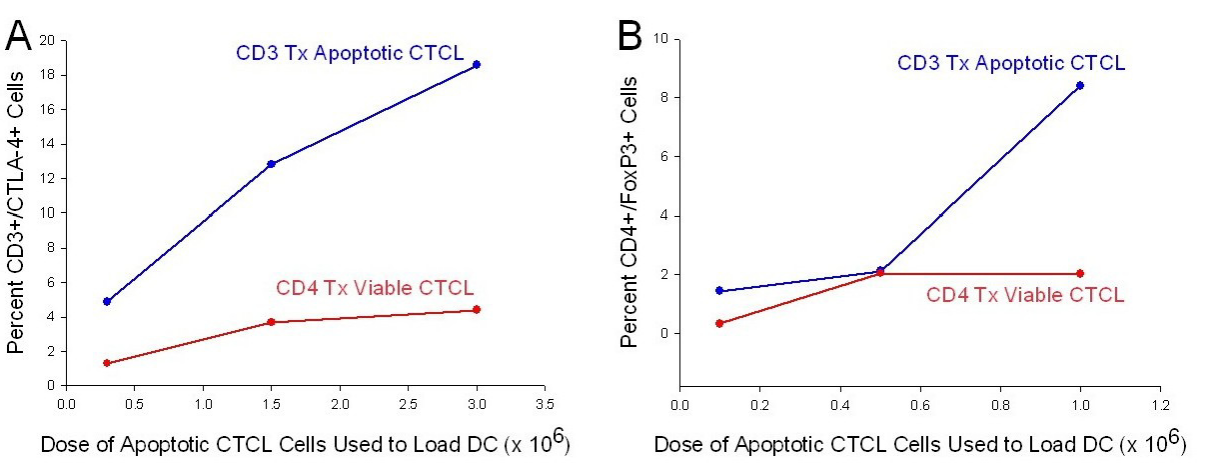
**Dose-response curve of Treg induction in CTCL cells**. **A. **Increasing numbers of CD3-treated apoptotic CTCL cells (0.3 × 10^6^, 1.5 × 10^6^, 3.0 × 10^6^) were added to a constant number of DC (10^5^), co-cultured overnight and used to stimulate freshly isolated autologous CTCL cells. As controls, increasing numbers of non-loaded DC were cultured with CD4-treated viable CTCL cells. Then additional CTCL cells were added and co-cultured overnight. The percentage of membrane CD3^+^/cytoplasmic CTLA-4^+ ^CTCL cells was measured by flow cytometry gated on the lymphocyte population. **B**. Increasing numbers of apoptotic CTCL cells (0.10 × 10^6^, 0.5 × 10^6^, 1.0 × 10^6^) were added to 10^5 ^DC and co-cultured overnight, followed by addition of 10^6 ^purified CTCL cells. The percentage of membrane CD4^+^/cytoplasmic FoxP3^+ ^expressing cells was measured by flow cytometry. Apoptotic = CD3-treated, Viable = CD4-treated

We performed a dose response curve by adding increasing numbers of CTCL cells that had been rendered apoptotic by incubation with CD3-antibody conjugated to magnetic beads to overnight cultures of column-generated transitioning DC. We found that in the presence of column-generated DC that had ingested apoptotic CTCL cells, the percentage of responding CTCL cells that expressed CTLA-4 increased as the number of apoptotic cells available for DC ingestion in the co-culture increased (Fig 4A). A dose of 0.3 × 10^6 ^apoptotic cells fed to 10^5 ^DC induced only 4.88% of the freshly added CTCL cells to express cytoplasmic CTLA-4. As the dose of apoptotic CTCL cells rose to 1.5 × 10^6 ^and 3.0 × 10^6^, the percentage of freshly added CTCL cells expressing CTLA-4 increased to 12.83% and 18.6% respectively. In contrast, as a control, addition of increasing numbers of viable CTCL cells to the constant number of DC did little to enhance the percentage of CTCL cells expressing CTLA-4. In addition, as the dose of apoptotic cells fed to a constant number of DC increased, the percentage of CD4^+ ^T cells expressing FoxP3, the phenotypic hallmark of Tregs, increased as well (Fig. 4B). At 0.1 × 10^6 ^apoptotic cells fed to a constant number of DC, the percentage of CTCL cells expressing FoxP3 was 1.45%, while a 10-fold increase in the apoptotic cell dose to 1.0 × 10^6 ^resulted in a 5.8-fold increase to 8.41% of CD4^+ ^T cells expressing FoxP3. This dose-dependent increase in FoxP3 expression was not seen in the control group where increasing numbers of CD4-treated viable CTCL cells were added to a constant number of DC. Therefore, the generation of CTLA4^+^/FoxP3^+ ^Treg cells may be controlled, to some degree, by the level of apoptotic cells fed to the DC.

### Treg CTCL cells suppress CD8-mediated perforin expression

**Figure 5:**
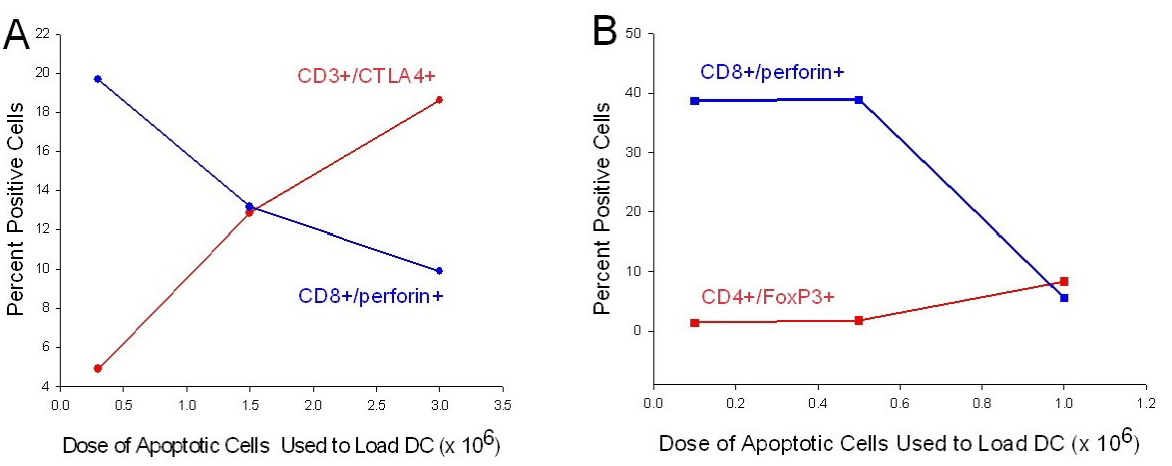
**Dose-dependent decrease in CD8-perforin expression as number of phenotypic Treg cells increase**. **A. **Increasing numbers of CD3-treated apoptotic CTCL cells (0.3 × 10^6^, 1.5 × 10^6^, 3.0 × 10^6^) were added to a constant number of DC (10^5^), co-cultured overnight and used to stimulate freshly isolated autologous CTCL cells and CD8^+ ^cells. As the percentage of membrane CD3^+^/cytoplasmic CTLA4^+ ^cells increased there was an inversely related decrease in the number of membrane CD8^+^/cytoplasmic perforin^+ ^cells. **B. **Increasing numbers of apoptotic CTCL cells (0.10 × 10^6^, 0.5 × 10^6^, 1.0 × 10^6^) were added to 10^5 ^DC and co-cultured overnight, followed by addition of 10^6 ^purified CTCL cells and CD8^+ ^cells. As the percentage of membrane CD4^+^/cytoplasmic FoxP3^+ ^cells increased, there was an inversely related decrease in the number of membrane CD8^+^/cytoplasmic perforin^+ ^cells. Apoptotic = CD3-treated, Viable = CD4-treated

As the percentage of CTCL cells that were stimulated to adopt a Treg phenotype increased, the percentage of CD8^+ ^T cells in co-culture expressing perforin, a granule that mediates CD8^+ ^effector T cell cytotoxicity [13], inversely decreased (Fig 5). The percentage of CD3^+^/CTLA4^+ ^cells was increased from 4.88% to 18.6% (Fig. 5A) in the presence of DC loaded with increasing numbers of apoptotic cells. Concomitantly, there was a reduction in the percentage of cells co-expressing CD8/perforin, from 19.68% down to 9.87% at the highest dose of apoptotic cells used to load the DC. Similarly, as the percentage of cells expressing CD4^+^/FoxP3^+ ^increased from 1.45% to 8.41% (driven by a high dose of apoptotic cell DC loading), the percentage of CD8^+ ^T cells expressing perforin dropped sharply from 36.69% to 5.58% (Fig. 5B), identifying a possible threshold of 1–1.5 × 10^6 ^apoptotic cells/10^5 ^DC required for induction of a Treg phenotype by the autologous DC. Therefore, when CTCL cells are induced to adopt a Treg phenotype by co-culturing them with autologous DC that have ingested large numbers of apoptotic CTCL cells, they display the phenotypic characteristics of Treg, a conversion that may mediate the reduction in the percentage of CD8 T cells that express perforin.

### Expansion of CD8 T cell compartment following co-culture with autologous DC that have ingested low numbers of apoptotic cells

**Figure 6:**
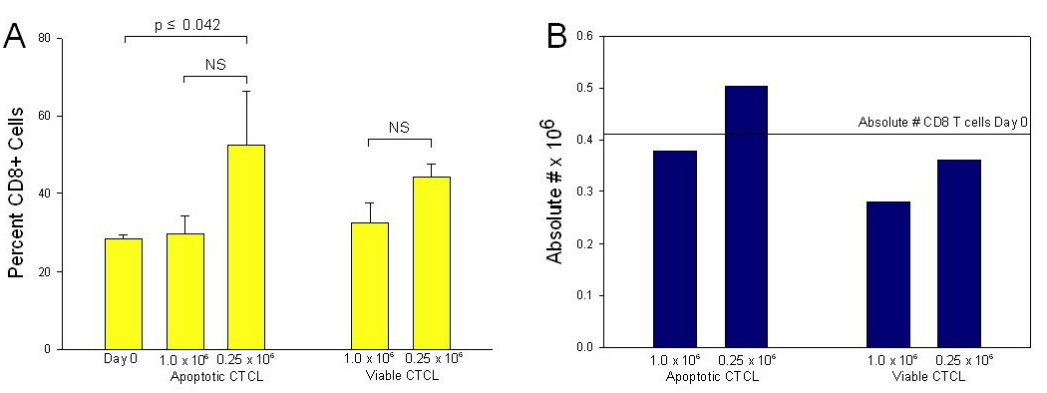
**Expansion of CD8 T cell compartment following co-culture with DC loaded with low numbers of apoptotic CTCL cells**. **A. **Autologous DC (10^6^) were co-cultured with 1.0 × 10^6 ^and 0.25 × 10^6 ^apoptotic CTCL cells, followed by addition of freshly isolated autologous CTCL cells and CD8^+ ^cells. Percentage of CD8^+ ^T cells in co-culture increased significantly upon co-culture with autologous DC loaded with 0.25 × 10^6 ^apoptotic cells. **B**. The absolute number of CD8^+ ^T cells increased upon co-culture with DC loaded with 0.25 × 10^6 ^apoptotic cells. Data analyzed using student's t test. Apoptotic = CD3-treated, Viable = CD4-treated, NS = Not significant.

We next sought to determine whether controlling the level of apoptotic T cells added to DC could shift the balance of suppression towards the generation of anti-tumor immunity by loading autologous DC with low numbers of apoptotic cells. To do this, autologous DC were fed both a high number of apoptotic CTCL cells (above the demonstrated level of Treg induction) and a low number of apoptotic CTCL cells, and expansion of the CD8 T cell population was monitored. The percentage of CD8 T cells increased by 83% in the group of DC fed low numbers of apoptotic T cells (p ≤ 0.042), as compared to control Day 0 samples (Fig. 6A). In addition, the absolute number of CD8 T cells recovered from overnight culture of column-passaged DC loaded with apoptotic CTCL cells also increased by 20% when compared to the initial Day 0 level of CD8 T cells (Fig. 6B). Expansion of the CD8 T cell compartment was greatest when DC were co-cultured with a lower number of apoptotic T cells, below the threshold for Treg induction, and this expanded CD8 T cell population might hold the potential to generate anti-tumor immunity.

### Increased perforin activity and increased T cell death following loading of autologous DC with low numbers of apoptotic cells

**Figure 7:**
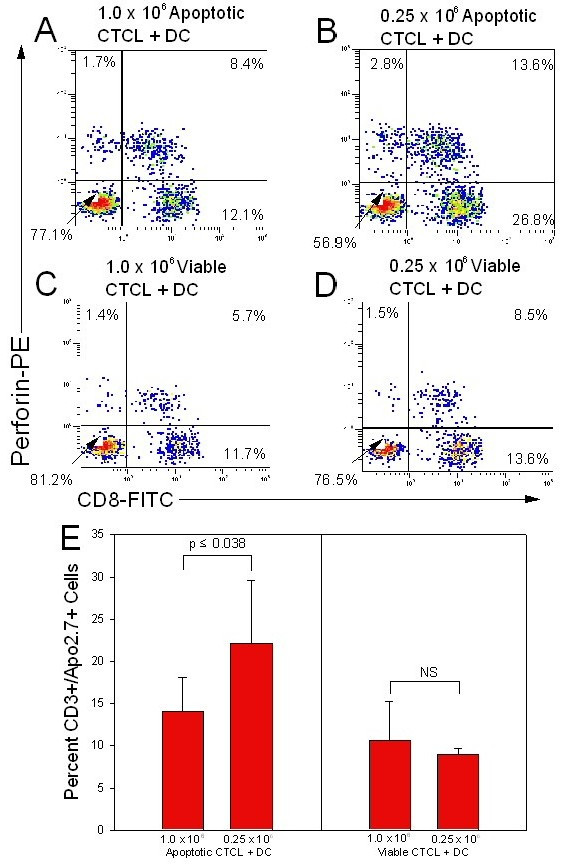
**Enhancement of CD8 T cell response upon exposure to autologous DC loaded with low numbers of apoptotic CTCL cells**. **A – D. **DC loaded with 1.0 × 10^6 ^and 0.25 × 10^6 ^apoptotic malignant T cells stimulated increased perforin activity in the CD8 T cell compartment, as compared to DC co-cultured with 1.0 × 10^6 ^and 0.25 × 10^6 ^viable CTCL cells. Results are presented as quadstats measuring the expression of membrane CD8/cytoplasmic perforin as determined by flow cytometric analysis of the gated lymphocyte population. Results are representative of 5 separate experiments performed on cells isolated from cultures of 5 different CTCL patients. **E. **Increased perforin activity in the CD8 T cell compartment translated into increased CTCL cell death as measured by membrane CD3/cytoplasmic APO 2.7 staining. Data analyzed using student's t test. Apoptotic = CD3-treated, Viable = CD4-treated, NS = Not significant.

The expanded CD8 T cell compartment found when freshly purified CD8^+ ^T cells were co-cultured with autologous DC that had been loaded with low numbers of apoptotic cells also demonstrated increased perforin activity. The percentage of CD8^+ ^T cells expressing perforin, the major cytotoxic granule by which effector CD8 cells mediate their anti-tumor effects, rose from 8.4% (Fig 7A) to 13.8% (Fig 7B) when we lowered the dose of apoptotic cells fed to the DC. This increase in perforin activity was not seen in the control population, where viable cells were co-incubated with autologous DC. In addition, the increased perforin activity translated into significantly increased T cell death (p ≤ 0.038) in the added CD3^+ ^CTCL T cell population when CD8 T cells were co-cultured with low numbers of apoptotic cells and DC (Fig 7E). Under these conditions, we found that 57% more CTCL cells were killed in comparison to the level of cell death obtained when DC were loaded with high numbers of apoptotic cells and used to prime CD8 T cells. No significant change in CTCL cell death was noted when viable CTCL cells were added to the DC. These results indicate that the expanded CD8 T cell population not only demonstrates increased perforin expression phenotypically, but is also functionally competent to mediate increased apoptosis in freshly added CTCL cells.

## Discussion

The development of more effective DC-based immunotherapy has been hampered by a number of complicating factors, ranging from the length of culture necessary for DC development in vitro as well as a lack of knowledge of the most appropriate stage of DC maturation required for effective anti-tumor vaccination [14]. In addition, the most appropriate antigen source for DC loading remains to be determined, with a variety of existing strategies including: purified peptides [15]; tumor-targeted viral vectors [16]; tumor/DC hybrids; apoptotic whole tumor cells; and tumor lysates having been attempted and resulted in substantial variability in long-term clinical responses [17].

Utilizing a magnetic bead column, we were able to generate potentially immunogenic DC in a fashion that offers several advantages over existing methodologies: 1) DC generated in this fashion can be obtained after a single overnight culture, far more rapidly than existing culture methods which can take up to 7 days; 2) Apoptotic whole cells are used as a source of tumor antigen, ensuring that the spectrum of CTCL antigenicity is incorporated into the DC instead of singular known antigens which may not be optimal or may be lost as the tumor de-differentiates; 3) Passage of monocytes through the column matrix induces the synchronized differentiation of a large population of monocytes into DC, allowing for the generation of a more robust source of APC displaying a spectrum of antigens upon re-injection; 4) The rapidity and ease of manipulating the cellular subpopulations of apoptotic CTCL cells and antigen-presenting cells allows the ability to easily optimize the cell populations and added factors in the hope of creating more effective clinical immunotherapy.

In these experiments, we sought to further our understanding of the conditions that favor the induction of Treg cells following exposure to autologous DC co-cultured with large numbers of apoptotic CTCL cells. The use of the magnetic bead column provides a new avenue for rapid generation of tumor-loaded DC because the variety of cellular components inherent in DC differentiation can be titrated to optimize dosing with this method.

In our studies, CTCL cells were incubated with anti-CD3 antibody prior to passage through the magnetic bead column, rendering the antigen-experienced malignant T cells apoptotic, as had been previously demonstrated. In addition, the use of anti-CD4 antibody allowed for separation of CD4^+ ^CTCL cells to be used as a control population while at the same time not altering the CTCL cells. The membrane perturbation inherent in monocyte passage through the column matrix physically stimulates their transition into DC [18]. DC differentiation was measured by both the reduction of expression of the monocyte marker CD14 and the increased expression of class II and CD86. In all instances, significant changes in the expression of these cell markers were found, indicating that the column passaged monocytes were differentiating into DC and that co-culture of the maturing DC with apoptotic malignant T cells further stimulated their entry into the DC pathway. Differentiation of monocytes utilizing membrane stimulation, as in passage through the column matrix, is advantageous in particular in the setting of cancer immunotherapy since it has been demonstrated that physical perturbation is one of the more effective means of cross-priming phagocytosed peptides into the MHC class I pathway, which is crucial in generation of a CD8 anti-tumor response [18].

Following the generation of potentially immunogenic DC using the magnetic bead column, we sought to determine if Treg conversion could be induced. By loading dendritic cells with large numbers of apoptotic cells, in a ratio of > 10 apoptotic cells to 1 DC, we were able to show that CTCL cells could be induced to assume some of the phenotypic and functional features of Treg cells. These findings correlate with the work of Berger *et al*, who has shown that co-culture of freshly purified CTCL cells with autologous DC loaded with large numbers of apoptotic CTCL cells can induce a Treg profile in the responding CTCL cell population, one that has phenotypic and functional characteristics associated with Treg cells [4]. Treg are a subset of T cells that comprise 5–10% of the peripheral T cell population, and are involved in the maintenance of peripheral tolerance and prevention of autoimmune disease, regulating the response to infectious agents, transplanted tissues, and self antigens [19-21]. Treg are characterized by their ability to suppress immune responses primarily by inhibiting normal T cell antigen driven proliferation [22,23]. Treg have been found in increased numbers in solid tumors and are currently seen as one of the major hurdles to beneficial clinical responses in existing immunotherapies [24]. Removal of the suppressive Treg population has been shown to improve the outcome of cancer immunotherapy in both mouse models and clinical trials in a number of malignancies [24].

As the dose of apoptotic CTCL cells fed to a constant number of DC increased, expression of the Treg-associated markers CTLA-4, CD25, and FoxP3 also increased concomitantly. In addition, there appeared to be a threshold ratio of apoptotic cell:DC that corresponded with induction of the Treg phenotype. As the apoptotic cell:DC ratio increased from 5:1 to 10:1, expression of the hallmark of Treg cells, FoxP3, increased nearly four fold, indicating that perhaps there may be some internal DC checkpoint related to the number of processed apoptotic cells that governs the generation of an immune response versus a suppressive one. While specific mechanisms of this process remain unclear, we can speculate that perhaps a CD8 T cell response is favored when low numbers of peptides are presented while induction of an immunosuppressive response requires a higher antigen burden and presentation of elevated levels of peptides in MHC class II. In our experiments, the induced phenotypic Tregs also displayed some functional capabilities, as they were able to suppress CD8-mediated perforin expression in co-culture; an observation, which if borne out in vivo, may explain some of the failures in generating anti-tumor immunity with existing immunotherapies. These findings are in concert with our previous studies which demonstrated that cultured CTCL cells can be stimulated to assume a Treg phenotype and function suppressing normal T cell driven immune responses when exposed to DC that have ingested apoptotic cells [4]. In our present studies, we demonstrate that, in addition, freshly isolated CTCL cells can be driven to adopt a Treg phenotype by overnight exposure to DC loaded with large numbers of apoptotic cells.

By establishing that Treg cells could be rapidly generated using this method, we then sought to determine if that delicate balance between the DC and surrounding numbers of apoptotic cells could be shifted in the other direction, towards stimulating a more vigorous immune response. By lowering the dose of apoptotic cells processed by a constant number of DC, we were able to induce significant expansion of the CD8 T cell compartment. This expanded CD8 T cell compartment not only contained a greater percentage of perforin expressing cells but also mediated increased T cell death in the added CTCL cell population. In terms of existing immunotherapies, these findings hold major clues into possible mechanisms for tumor survival following treatment. If too aggressive an attempt is made at killing tumor cells in hopes of loading DC with large number of tumor antigens, it may lead to Treg generation and suppression of the desired immune response. A considerable body of literature exists supporting the idea that the difference between the DC's ability to induce tolerance or mediate immunity may be determined by its persistent exposure to antigen. [25,26] In our experiments, the DC that were exposed to larger numbers of apoptotic cells and hence had more persistent antigen exposure induced suppression of the CD8 T cell population. These findings support earlier work and help to reinforce the role of apoptosis in the induction of immunosuppression. [27,28]

A better understanding of the role of Treg cells in immunotherapy is crucial because while they hamper the effectiveness of cancer immunotherapy [24], Treg may be of great value for the therapy of autoimmune disease and transplantation tolerance. [21,29] The ability to use rapid methods such as the magnetic bead column allows for optimization of existing methodologies so that we can improve our understanding of apoptotic T cell and DC interactions, with the goal of creating more effective cancer immunotherapy.

## Competing interests

Drs. Berger, Edelson, and Yale University hold patents pertaining to the Transimmunization procedure.

## Authors' contributions

JDS and JGV carried out the experiments presented in this manuscript. CLB and RLE have defined the preliminary observations upon which this manuscript is based and provided intellectual guidance and supervision for the reported work and manuscript.
